# Diagnostic yield of patients with undiagnosed intellectual disability, global developmental delay and multiples congenital anomalies using karyotype, microarray analysis, whole exome sequencing from Central Brazil

**DOI:** 10.1371/journal.pone.0266493

**Published:** 2022-04-07

**Authors:** Ana Julia da Cunha Leite, Irene Plaza Pinto, Nico Leijsten, Martina Ruiterkamp-Versteeg, Rolph Pfundt, Nicole de Leeuw, Aparecido Divino da Cruz, Lysa Bernardes Minasi

**Affiliations:** 1 Genetics and Molecular Biology Graduate Program, Federal University of Goiás, Goiânia, Goiás, Brazil; 2 Replicon Research Group, Master Program in Genetics, School of Medical and Life Science, Pontifical Catholic University of Goiás, Goiânia, Goiás, Brazil; 3 Department of Human Genetics, Radboud University Medical Center, University Nijmegen Medical Centre, Nijmegen, Netherlands; 4 Human Cytogenetics and Molecular Genetics Laboratory, State Health Secretary of Goiás, Pontifical Catholic University of Goiás, Goiânia, Goiás, Brazil; University of Colorado Denver - Anschutz Medical Campus, UNITED STATES

## Abstract

Intellectual Disability (ID) is a neurodevelopmental disorder that affects approximately 3% of children and adolescents worldwide. It is a heterogeneous and multifactorial clinical condition. Several methodologies have been used to identify the genetic causes of ID and in recent years new generation sequencing techniques, such as exome sequencing, have enabled an increase in the detection of new pathogenic variants and new genes associated with ID. The aim of this study was to evaluate exome sequencing with analysis of the ID gene panel as a tool to increase the diagnostic yield of patients with ID/GDD/MCA in Central Brazil, together with karyotype and CMA tests. A retrospective cohort study was carried out with 369 patients encompassing both sexes. Karyotype analysis was performed for all patients. CMA was performed for patients who did not present structural and or numerical alterations in the karyotype. Cases that were not diagnosed after performing karyotyping and CMA were referred for exome sequencing using a gene panel for ID that included 1,252 genes. The karyotype identified chromosomal alterations in 34.7% (128/369). CMA was performed in 83 patients who had normal karyotype results resulting in a diagnostic yield of 21.7% (18/83). Exome sequencing with analysis of the ID gene panel was performed in 19 trios of families that had negative results with previous methodologies. With the ID gene panel analysis, we identified mutations in 63.1% (12/19) of the cases of which 75% (9/12) were pathogenic variants,8.3% (1/12) likely pathogenic and in 16.7% (2/12) it concerned a Variant of Uncertain Significance. With the three methodologies applied, it was possible to identify the genetic cause of ID in 42.3% (156/369) of the patients. In conclusion, our studies show the different methodologies that can be useful in diagnosing ID/GDD/MCA and that whole exome sequencing followed by gene panel analysis, when combined with clinical and laboratory screening, is an efficient diagnostic strategy.

## Introduction

Intellectual Disability (ID) is a complex, heterogeneous and multifactorial neurodevelopmental disorder characterized by cognitive impairment and difficulties in adaptive behaviour. ID can occur in isolation or associated with other clinical conditions beginning before the age of 18. The worldwide prevalence of children and adolescents with ID is estimated at around 3% [1, 2]. The genetic etiology of ID may include chromosomal abnormalities, submicroscopic chromosomal rearrangements, copy number variations (CNVs), and gene mutations. To date, a myriad of mutations in more than 1,000 genes have been associated with ID phenotypes [1, 3, 4].

Several technologies have been used in an attempt to identify the genetic causes of ID. For many years, the karyotype has been the gold standard for detecting numerical and/or structural chromosomal alterations with ≥5–10Mb in size. Chromosome Microarray Analysis (CMA) has become the first-tier clinical test to detect CNVs in patients with ID, global developmental delay (GDD), autism spectrum disorder (ASD), and Multiple Congenital Anomalies (MCA). However, despite of all available technologies, around 50% of cases remain undiagnosed [5–7].

Next Generation Sequencing (NGS) has been shown to be efficient in revealing new gene mutations and discrete genomic variations thereby increasing the genetic diagnostic yield of ID. NGS has become a powerful tool to aid in the elucidation of the genetic etiology of ID in a wide range of clinical applications and scenarios [8]. When used in pediatric populations with neurodevelopmental disorders, exome sequencing provides a molecular diagnostic yield of nearly 25% and has been progressively applied for molecular diagnosis in clinical settings [6]. Additionally, WES can be indicated for patients with genetic disorders and unspecific clinical characteristics and multiple genetic conditions, allowing less time “on the odyssey” in search of a definitive diagnosis [9, 10].

The putatively positive effect of different technical strategies for the genetic diagnosis in a Brazilian ID cohort would provide a new perspective of diagnosis that could lead to the inclusion of these technologies in the protocols of the Brazilian Public Health System, which is responsible for regulating the use of genetic testing nation-wide. Herein, we report the results of a study designed to evaluate the WES with analysis of the ID gene panel as a tool to increase the diagnostic yield of ID in a cohort of patients from Central Brazil with Intellectual Disability/Global Developmental Delay/Multiple Congenital Anomalies in addition to G-band Karyotyping and CMA approaches.

## Materials and methods

### Sampling

This is a retrospective cross-sectional study, from 2013 to 2017, which included a representative subset of a population composed by 369 patients with ID, GDD with or without MCA. The patients included in the study were physically examined and clinically diagnosed with ID, GDD or MCA by assistant physicians from the state public health service of Goiás. Subsequently, were referred to Replicon Research Group and the Laboratory of Human Cytogenetic and Molecular Genetics for karyotyping and CMA testing. The efficient screening of patients by the attending physicians and by the team of geneticists of the laboratories increased the number of patients assisted by our laboratory. In addition, our laboratory is the only public genetic services from Goiás, so most patients who need to undergo diagnostic genetic tests are referred to us.

Subsequently, a subset of cases with uneventful karyotype and CMA results were sent to the Genome Diagnostics Laboratory at the Radboud University Medical Center in Nijmegen, the Netherlands to be further tested by WES using an ID gene panel. The study was approved by the Research Ethics Committee from PUC Goiás, Brazil, under protocol code number 3.205.591. The patients’ parents voluntarily signed an informed consent form approved by the local Ethics Committee. The study was performed under the guidelines of the Declaration of Helsinki.

In the current study, a loss of follow-up for a subset of patients was observed over time, especially among those with a normal karyotype and CMA. We tried to recontact those families to explain about the risks and benefits of genetic testing. However, contacting was unsuccessful for a group of patients, mostly due to changes in the family’s telephone number, residency address, death of the child, and / or death of a biological parent. Moreover, some families simply declined the invitation to participate in the current study and refused to sign an informed consent. So, they were excluded from the cohort undergoing gene testing with a panel especially designed for ID, GDD with or without MCA.

### Karyotyping and chromosomal microarray analysis

Peripheral blood samples were used for cytogenetic analyses. GTG banding at 550 bands was performed for all patients following standardized procedures, and chromosome analyses were done using the IKAROS^®^ software (Metasystems Corporation, Altlussheim, Germany).

Genomic DNA was isolated from the peripheral blood samples using Illustra Blood Genomic Prep Mini Spin Kit (GE Healthcare Life Sciences, Piscataway, New Jersey, USA), following the manufacturer’s instructions. The CMA was carried out on patients whose karyotype showed no numerical and structural alterations. CMA was also was performed in the biological parents of all cases in order to establish the origin of rearrangements. In the current study, the GeneChip^®^ CytoScanHD^™^ array (ThermoFisher Scientific, USA) was the array of choice due to its excellent coverage of the human genome with 1.9 million non-polymorphic probes combined with 750,000 SNP probes. CMAS was carried out according to the manufacturer’s recommendations. Chromosomal analyses were performed using the Chromosome Analysis Suite 3.0 (ChAS^®^) software (ThermoFisher Scientific, USA) based on the genome reference hg19/GRCh37, using a filter with 50 markers for gains and 25 markers for losses both with size ≥ 100 kb. CNVs were classified according to their nature, based on previously published international consensus and guidelines [11–14].

For both technologies the results were reported according to the International System for Human Cytogenomic Nomenclature [15].

### Whole Exome Sequencing (WES) followed by target gene panel analysis

The target gene panel (TGP) analysis was performed on cases who had not received a diagnosis from either karyotype or CMA and their biological parents. Isolated DNA samples were sent for exome sequencing followed by TGP analysis according to De Ligt and colleagues [16]. The intellectual disability gene panel encompassing 1,252 genes (version DG-2.16) available at Diagnostics Nijmegen Laboratory and based on the last genome build reference available (hg19/GRCh37) and exome wide CNV analysis. Preparation and enrichment of genomic DNA were done using Agilent SureSelectXT Human All Exon 50Mb kit. Exome sequencing was performed on the HiSeq 2500 System platform (Illumina, USA), providing 20x coverage of > 94% of targeted bases. The variants found were classified according to the Association of Clinical Genetic Laboratory Diagnostics (VKGL) and Association for Clinical Genetic Science (ACGS) [17].

## Results

**Fig 1:**
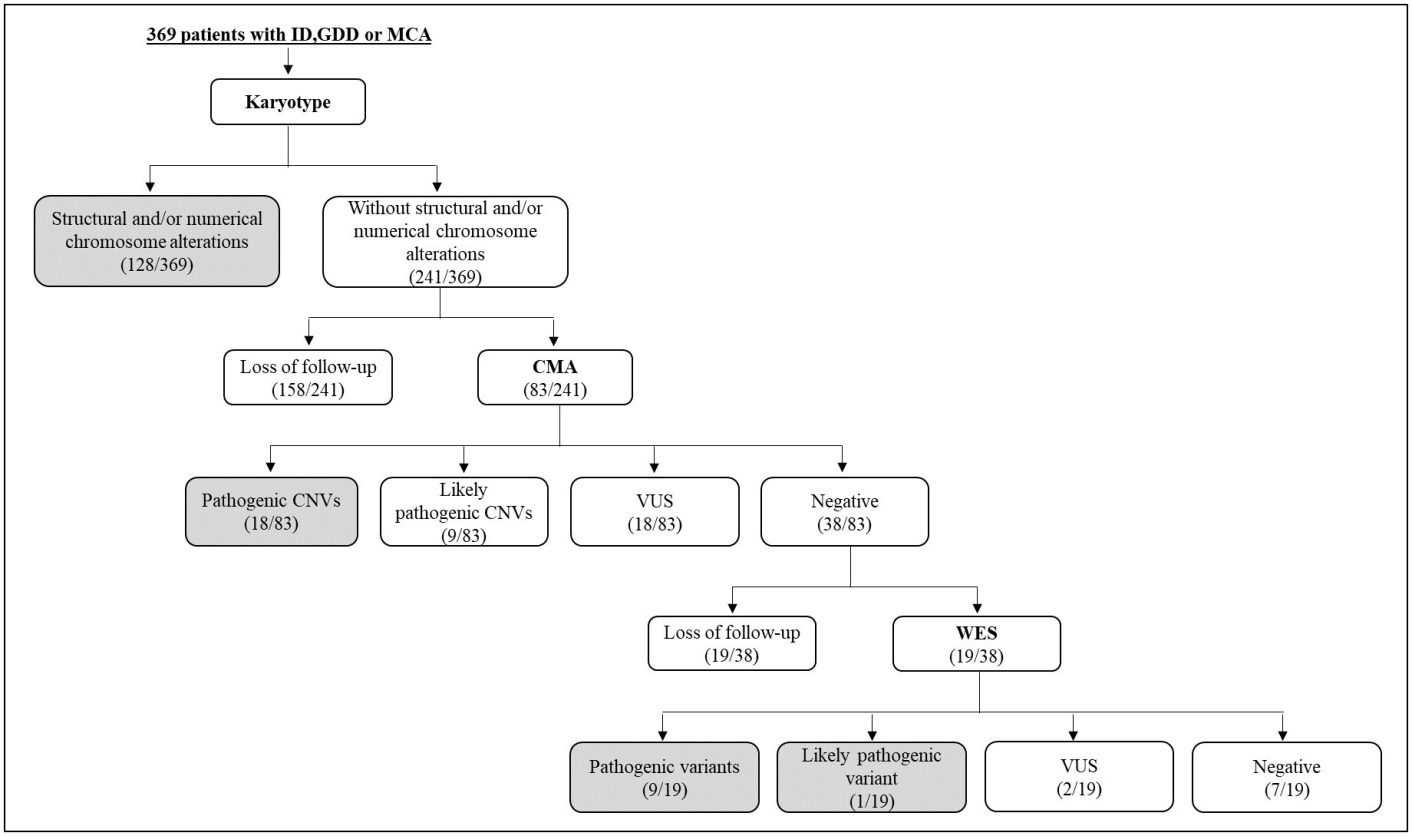
Diagram of the testing workflow and number of cases in each step for the genetic diagnosis of ID, GDD and MCA from Central Brazil. The highlighted rectangles indicate the total of cases for which diagnoses were reached.

The cohort of 369 patients comprised 52.6% (194/369) females and 47.4% (175/369) males with ID/GDD with or without MCA. Of these, 93.2% (344/369) were under the age of 18 years old, while 6.8% (25/369) were ≥ 18 years old. The Fig 1 shows the testing workflow and the number of patients in the study.

GTG banding karyotypes of 34.7% (128/369) patients showed structural and/or numerical alterations, of which the vast majority, 81.2% (104/128), were diagnosed with Down Syndrome. The remainder 65.3% (241/369) of cases showed no visible aberration in their karyotypes.

After initial karyotype screening, CMA was not performed in 42.8% (158/369) of the patients due to loss of follow-up in the cohort. Therefore, CMA was performed in 83 patients with no visible alterations in their karyotype. The reports included pathogenic CNVs in 21.7% (18/83) patients, 10.8% (9/83) of patients presented likely pathogenic CNVs, 21.7% (18/83) of patients exhibited variant of uncertain significance (VUS) CNVs, and 45.8% (38/83) of patients showed no detectable alterations in the CMA (S1 Table).

The TGP analysis on exome data was performed in 50% (19/38) patients without a diagnosis from either GTG-banding or CMA and their biological parents. This was not possible for the other half of the patients due to loss of follow-up. We identified variants in an intellectual disability gene in 63.1% (12/19) of patients. The variants correspond to 75% (9/12) pathogenic variants, 8.3% (1/12) likely pathogenic variant and 16.7% (2/12) VUS. Of the total of variants identified, 75% (9/12) were *de novo* variants and 25% (3/12) presented inherited variants from an unaffected carrier parent. The most prevalent variants were *missense* variants (66.7%), followed by *nonsense* variants (16.7%), a *frameshift* variant (8.3%), and a copy number loss (8.3%) (Table 1).

**Table 1:** Summary of the molecular results of cases with ID/GDD/MCA investigated with a target gene panel exome sequencing.

Case	Sex	Gene	Genomic position	Inheritance	Mutation type	Origin	Zygosity	Classification of variants	Syndrome	#OMIM
001	F	*MECP2*	ChrX(GRCh37):g.153296082_153296116del; NM_004992.3:c.1163_1197del; p.(Pro388fs)	X-linked	Frameshift	*de novo*	Heterozygous	Pathogenic	Rett Syndrome	312750
002	F	*DDX3X*	ChrX(GRCh37):g.41202509T>G; NM_001193416.2:c.584T>G; p.(Ile195Ser)	X-linked	Missense	*de novo*	Heterozygous	Pathogenic	Intellectual developmental disorder, X-linked, syndrome	300958
003	M	___	Negative*	___	___	___	___	___	___	___
004	M	___	Negative*	___	___	___	___	___	___	___
005	M	*TRAPPC9*	Chr8(GRCh37):g.140744221C>T; NM_031466.6:c.3573+1G>A (r.spl.?)	AR	Nonsense	Inherited Pat	Heterozygous	Likely pathogenic	Mental retardation, autosomal recessive 13; MRT13	613192
			Chr8(GRCh37):g.141285764del; NM_031466.6:c.2565del (p.(Thr856fs))			Inherited Mat				
006	F	*SOS1*	Chr2(GRCh37):g.39249914C>T; NM_005633.3:c.1655G>A (p.(Arg552Lys))	AD	Missense	*de novo*	Heterozygous	Pathogenic	Noonan Syndrome 4	610733
007	M	*DNMT3A*	seq[GRCh37] del(2)(p23.2p23.2)dn Chr2:g.(25387621_25457148)_(25462167–25462322)del	AD	CNV partial Loss	*de novo*	Heterozygous	Pathogenic	Tatton-Brown–Rahman Syndrome	615879
008	F	___	Negative*	___	___	___	___	___	___	___
009	M	*NALCN*	Chr13(GRCh37):g.101881844A>G; NM_001350748.1:c.1526T>C (p.(Leu509Ser))	AD	Missense	*de novo*	Heterozygous	Pathogenic	Congenital contractures of the limbs and face, hypotonia, and developmental delay	616266
010	M	*PTPN1*	Chr12(GRCh37):g.112915455T>C; NM_002834.4; c.854T>C; p.(Phe285Ser)	AD	Missense	*de novo*	Heterozygous	Pathogenic	Noonan Syndrome—1	163950
011	F	___	Negative*	___	___	___	___	___	___	___
012	M	*MED12*	ChrX(GRCh37):g.70348547A>G; NM_005120.2:c.3454A>G (p.(Ile1152Val))	X-linked	Missense	Inherited Mat	Hemizygous	VUS	* *	___
013	M	___	Negative*	___	___	___	___	___	___	___
014	F	___	Negative*	___	___	___	___	___	___	___
015	F	*PPP1CB*	Chr2(GRCh37):g.28999810C>G; NM_206876.1:c.146C>G; p.Pro49Arg	AD	Missense	*de novo*	Heterozygous	Pathogenic	Noonan syndrome-like disorder with loose anagen hair 2	617506
016	M	*SYT1*	Chr12(GRCh37):g.79842738T>C; NM_001135805.1:c.1103T>C (p.(Ile368Thr))	AD	Missense	*de novo*	Heterozygous	Pathogenic	Baker-Gordon Syndrome	618218
017	F	*SYNGAP1*	Chr6(GRCh37):g.33409095C>T; NM_006772.2:c.2059C>T (p.(Arg687*))	AD	Nonsense	*de novo*	Heterozygous	Pathogenic	Mental retardation, autosomal dominant 5; MRD5	612621
018	F	___	Negative*	___	___	___	___	___	___	___
019	M	*FLNA*	ChrX(GRCh37):g.153581453T>G; NM_001456.3:c.6118A>C; p.(Ser2040Arg)	X-linked	Missense	Inherited Mat	Hemizygous	VUS	**	___

___ = not applicable; AR = autosomal recessive; AD = autosomal dominant; CNV = copy number variation; Mat = maternal; Pat = paternal; VUS = variant of uncertain significance.

* Negative means that no mutation was found using gene panel exome sequencing.

**At present, no syndrome has yet been associated with the identified variant.

The diagnostic yields achieved for the three different tests were 34.7% (128/369), 21.7% (18/83), and 52.6% (10/19) for karyotype, CMA, and WES, respectively. To reach a diagnosis, for CMA only pathogenic variants we considered, while for WES, pathogenic and likely pathogenic variants were considered. In the current study, combining a clinical screening and three different genomic methodologies, we obtained an overall diagnostic rate of 42.3% (156/369) for the ID/GDD/MCA cases.

## Discussion

In our cohort of 369 patients with ID/GDD/MCA, karyotyping was efficient to identify numerical and/or structural alterations in nearly 1:3 patients. This high diagnostic rate observed in our cohort could be due to the effective clinical triage of our ID patients and the fact that prenatal diagnoses is rarely done throughout the public health system in Brazil. Moreover, Down Syndrome (DS) was the most prevalent aneuploidy in our cohorts, consistent with several other previous observations that DS is responsible for the largest proportion of chromosomal findings reported by the karyotype [18], which has also been the leading cause of this phenotypic trait among children [19–21].

Despite its limited resolution, the G-banded karyotype has been the gold standard for detecting genetic rearrangements in patients with ID/GDD for over 35 years [22]. GTG banding is considered the first-tier test performed in individuals who present with craniofacial dysmorphisms or syndromic features and families who have a history of chromosomal disorders or recurrent miscarriages [23]. Regardless of this methodology being continuously used in patients with ID/GDD, our results showed a significant percentage (65.3%) of patients without numerical and/or structural aberrations, indicating the limited usefulness of this methodology, especially because of the heterogeneous aetiology of ID/GDD, which depends on populational variations, disease classification and the availability of diagnostic facilities. Nevertheless, as pointed out by Sadek and Mohamed [18], the G-banded karyotyping remains a useful tool with ID/GD with or without dysmorphic traits, especially in countries with limited resources and unequal access to the public service providers.

Since 2010, the CMA has been considered the first-tier clinical diagnostic approach for individuals with idiopathic ID/GDD, ASD, and / or MCA due to its capacity to detect CNVs across the genome with ten times greater resolution and higher diagnostic yield than GTG banding [11, 22, 24]. In the current study, CMA alone yielded a proper diagnosis for approximately 1:5 children with ID/GDD/MCA, which has been in accordance to previous studies [11, 22, 25, 26].

However, in 67.4% of patients, we could not determine the genomic alterations that could explain patients’ ID/GDD/MCA. Considering this, we aimed for NGS technologies as they have been reported useful in clinical practices and provide a substantial opportunity to improve the diagnosis in ID/GDD [27].

The TGP analysis using exome sequencing data was performed in 19 trios after no karyotype and CMA alterations were found. The ID gene panel lead to a diagnostic yield of 52.6%, where twelve affected individuals have pathogenic, likely pathogenic, and uncertain clinical significance variants in the following genes: *DDX3X*, *DNMT3A*, *FLNA*, *MECP2*, *MED12*, *NALCN*, *PPP1CB*, *PTPN11*, *SOS1*, *SYNGAP1*, *SYT1*, and *TRAPPC9*. Based on our findings, we associated these mutations with the patient’s phenotypes, and the approach demonstrated itself as a powerful method to enhance the diagnostic yield of cases harboring traits of ID/GDD.

It is important to note the high heterogeneity found in our cohort, where each case presented a (likely) pathogenic variant in a different gene, resulting in 12 different mutated genes. We also observed a high rate of *de novo* mutations among pathogenic variants, corresponding to 75% (9/12) of the cases, corroborating previously reports that a significant number of mutations in ID/GDD patients are *de novo* events [1, 24, 28–31].

Furthermore, 25% of individuals were males with autosomal recessive or X-linked variants inherited from an unaffected carrier parent. Two male patients had a maternally inherited variant, one patient with a *MED12* gene variant, and another a variant in the *FLNA* gene. Variants in the *MED12* gene have been described as associated with X-linked disorders such as Opitz-Kaveggia Syndrome [OMIM #305450], Lujan-Fryns Syndrome [OMIM #309520], and Ohdo Syndrome, X-linked [OMIM #300895] [32]. The *MED12* gene-specific variant found in our patient has not been reported before in the literature nor in the Genome Aggregation Database (GnomAD) (http://gnomad.broadinstitute.org) and in the DatabasE of genomiC varIation and Phenotype in Humans using Ensembl Resources (DECIPHER). Also, the pathogenicity and clinical relevance of this variant are as yet unclear. Pathogenic variants in the *FLNA* gene [OMIM *300017] are associated with numerous syndromes with broadly variable clinical features. Thus, it is unclear whether this variant found in our patient is pathogenic. Segregation analysis in maternal family members for both patients’ families would help to further determine the pathogenicity and clinical relevance of these variants.

In another male patient born to non-consanguineous parents we identified a homozygous pathogenic variant in the *TRAPPC19* gene inherited from both his mother and father who are heterozygous for this variant. This pathogenic variant has already been described as causative for mental retardation, autosomal recessive 13 [OMIM #613192].

Moreover, additional interesting finding was an interstitial heterozygous loss of ~5Kb in 2p23.2 that was not identified by CMA approach because of its small size. This deletion results in the loss of the last four exons of the *DNMT3A* gene. A similar partial deletion involving the last three exons was described by Hamdan and colleagues [33]. Together, these results shed light about the importance to report variants that have not been reported before and the attention for small copy number variants that can cause haploinsufficiency.

Using the intellectual disability gene panel on exome data, with great sensitivity and specificity, allowed us genotype-phenotype correlations for different genes related to ID/GDD/MCA. However, the genetic diagnostic was not reached for 36.8% of patients in our study. From them, whole genome or whole exome sequencing could be the next step, despite the possibility of failing to arrive at the genomic diagnosis due to the complexity and heterogeneity of ID/GDD/MCA.

Several cohorts have been described the diagnostic yield of target exome sequencing in individuals with ID ranging from 21% - 55.7%. Pekeles and colleagues [30] conducted a study with a cohort of 48 patients using four different panels and obtained a diagnostic rate of 21%. Yamamoto and colleagues [34] with a cohort of 133 patients obtained a diagnosis rate of 29.3%. In a study with 4.813 genes and 106 patients, Gieldon and colleagues [35] reports a diagnostic rate of 34%. Stojanovic and colleagues [36] in their study with 88 children with moderate to severe ID / GDD obtained a diagnostic rate of 55.7% using a panel with 4.813 genes. Other studies based on WES or WGS suggest a diagnostic rate ranging between 8% to 60% depending on the selection criteria [37]. According to Martínez and colleagues [38], the similar diagnostic yield between target gene panel and WES/WGS would make these methodologies equivalent from the diagnostic point of view.

Overall, due to combination of clinical and methodological screening for ID, we had a diagnostic rate of 42.3% (156/369) with a net yield of 96.3% (185/192) as for those who agreed to perform the three genetic tests only 7 cases remained undiagnosed. Our study highlights the limitation of the Brazilian Public Health System, especially in the scenario of intellectual diseases. Many patients do not receive their molecular diagnosis due to the loss of follow-up or even a lack of unified medical records. Thus, our results revealed the urgency for a better organization of public health systems to reduce the loss of follow-up in the cohort of people with ID/GDD. We also emphasize that the combined use of GTG karyotyping, CMA, and TGP for the ID/GDD diagnosis was appropriate and cost-effective. However, the limited sample size and patients’ loss during the study might weaken our conclusions. Further studies should be conducted with a larger cohort of patients to add strength to the conclusions in order to establish a national protocol for molecular diagnosis of ID/DGG patients in Brazil under the act and policies of the national Unified Health System.

In conclusion, the systematic combination of different methodologies proved useful for the genetic diagnoses of ID/GDD especially in scenarios of public health care where access to the services is limited by lack of equity and funds are scarce. We highlight the TGP proved to be an efficient strategy, with a reasonably high yield of in undiagnosed ID/GDD patients, higher than previous reports [29]. Our results showed that TGP should be considered a second-tier powerful strategy for the diagnosis of cases with ID/GDD after a negative result of CMA and prior to the analysis of whole exome. Moreover, following these steps, besides been cost-effective for developing countries, it would prevent the identification of genes considered medically unactionable especially in countries where decision-analytic policy models aren’t fully developed and would minimize the negative ethical impacts and improve patient–provider communication and shared decision making. On the other hand, we accentuate the importance of adequately choosing the target genes for a specific ID/DGG panel because with the advances of sequencing technology new ID/DGG genes are being constantly identified and should be aggregated into the panels. Finally, adequate clinical and laboratory screening, helped not only to elucidate the genetic etiology of ID/GDD/MCA, but also improved familial non-directive genetic counselling in a public health service setting.

## Supporting information

S1 Table Clinical and molecular features of patients investigated with CMA. (PDF)
