# Supplementary material for: Diagnostic yield of patients with undiagnosed intellectual disability, global developmental delay and multiples congenital anomalies using karyotype, microarray analysis, whole exome sequencing from Central Brazil
Source: PLoS One. 2022 Apr 7;17(4):e0266493. doi: 10.1371/journal.pone.0266493 (PMC8989190; doi:10.1371/journal.pone.0266493)
Supplement: S1 Table — (PDF) [file pone.0266493.s001.pdf]

Supplementary table - Clinical and molecular features of patients investigated with CMA.

Case	Clinical features*	Age (yo)	Sex	CNV	Size (Mb)	Microarray nomenclature	Selected OMIN Morbid Genes**	Origin	Interpretation	Syndrome	#OMIM
001	GDD, SS	9	F	NAF	***	***	***	***	Negative	***	***
002	GDD	11	M	Gain	3.68	arr[GRCh37] 17p11.2(16,769,800_20,446,820)x3 dn	*COPS3, SMCR9, RAI1, SMCR5, TOM1L2, LRRC48, ATPAF2, DRG2, MYO15A, ALKBH5, FLII, SMCR8, SHMT1, USP32P2, CCDC144B, B9D1,MFAP4, RNF112*	*de novo*	Pathogenic	Potocki-Lupski syndrome	610883
003	GDD	17	M	Gain	0.67	arr[GRCh37] 7q31.32(122,366,542_123,036,250)x3 pat	*CADPS2*	Inherited pat	VUS	***	***
004	GDD, SS, MS	2	M	Gain	0.52	arr[GRCh37] 12q13.13q13.2(54,462,464_54,980,062)x3 dn	***	*de novo*	VUS	***	***
005	GDD, MS	9	M	NAF	***	***	***	***	Negative	***	***
006	GDD	11	F	Loss	4.18	arr[GRCh37] Xq27.3q28(144,580,614_148,757,072)x1 dn	*CXorf1/TMEM257, FMR1, TMEM185A, IDS*	*de novo*	Pathogenic	Fragile X syndrome	300624
007	GDD, MS, MCA	4	F	Gain	59.41	arr[GRCh37] 18q11.1q23(18,608,373_78,014,123)x2-3 dn	18q Partial Trisomy	*de novo*	Pathogenic	ID/MCA non syndromic	***
				Gain	25.72	arr[GRCh37] Xp22.33p21.3(168,546_25,887,307)x3 dn	*NLGN4X, AP1S2, NHS, CDKL5, RPS6KA3, MBTPS2, SMS, ARX*	*de novo*	Pathogenic		
008	GDD, SS	25	F	NAF	***	***	***	***	Negative	***	***
009	GDD, MS, ALS	10	F	NAF	***	***	***	***	Negative	***	***
010	GDD, MS	9	F	NAF	***	***	***	***	Negative	***	***
011	GDD, BD, MS	8	F	Loss	10.89	arr[GRCh37] 1p31.3p31.1(68,693,129_79,580,916)x1 dn	*AK5*	*de novo*	Pathogenic	ID/MCA non syndromic	***
012	GDD	6	M	NAF	***	***	***	***	Negative	***	***
013	GDD	16	M	Gain	1.43	arr[GRCh37] 7q11.23(72,718,277_74,147,166)x3 dn	*TRIM50, FKBP6, FZD9, BAZ1B, BCL7B, TBL2, MLXIPL, VPS37D, WBSCR27, WBSCR28, ELN, LIMK1, EIF4H, LAT2, CLIP2, GTF2IRD1, GTF2I*	*de novo*	Pathogenic	WBS Duplication syndrome	609757
014	GDD, SS	14	M	Gain	0.34	arr[GRCh37] 22q11.23q12.1(25,656,237_25,994,326)x3 mat	***	Inherited mat	LP	Chromosome 22q11.2 duplication syndrome	608363
015	GDD, MS	8	F	Gain	0.14	arr[GRCh37] Xq28(152,720,466_152,860,955)x3 mat	*ATP2B3*	Inherited mat	VUS	***	***
016	GDD, MS	5	F	NAF	***	***	***	***	Negative	***	***
017	GDD	8	F	NAF	***	***	***	***	Negative	***	***
019	ALS	8	M	Gain	0.12	arr[GRCh37] Xp22.33(390,150_515,617)x3 dn	***	*de novo*	VUS	***	***
020	ALS	6	M	NAF	***	***	***	***	Negative	***	***
021	GDD	1	M	Gain	0.36	arr[GRCh37] Xq28(153,792,708_154,149,474)x2 mat	*DKC1*	Inherited mat	LP	Hoyeraal-Hreidarsson Syndrome	305000

Supplementary table - Clinical and molecular features of patients investigated with CMA. **(cont.)**

Case	Clinical features*	Age (yo)	Sex	CNV	Size (Mb)	Microarray nomenclature	Selected OMIN Morbid Genes**	Origin	Interpretation	Syndrome	#OMIM
022	GDD	12	F	Loss	0.75	arr[GRCh37] 22q11.21(20,716,902_21,465,662)x1 pat	*ZNF74, SCARF2, MED15, SNAP29, CRKL, LZTR1, SLC7A4*	Inherited pat	LP	DiGeorge Syndrome	188400
023	SS, GDD, MS, MCA	1	F	Loss	3.86	arr[GRCh37] 4p16.3(68,345_3,926,333)x1 dn	*ZNF141, IDUA, LETM1, WHSC1, NELFA/WHSC2*	*de novo*	Pathogenic	Wolf-Hirschhorn Syndrome	194190
				Loss	1.55	arr[GRCh37] 4p16.3p16.2(4,177,795_5,724,404)x1 dn	*MSX1, STK32B, C4orf6, EVC2, EVC*	*de novo*	Pathogenic		
				Gain	4.21	arr[GRCh37] 9p24.3p24.2(203,861_4,416,073)x3 dn	*DOCK8, KANK1, SMARCA2, VLDLR*	*de novo*	Pathogenic		
024	GDD	3	M	Loss	15.35	arr[GRCh37] 4q34.1q35.2(175,611,686_190,957,473)x1 dn	*GLRA3, TRAPPC11*	*de novo*	VUS	***	***
025		9	M	NAF	***	***	***	***	Negative	***	***
026	GDD,MS,MCA	6	F	Loss	15.39	arr[GRCh37] 13q32.3q34(99,712,845_115,107,733)x1 dn	*ZIC2, PCCA, NALCN, FGF14, LIG4, COL4A1, COL4A2, UPF3A*	*de novo*	Pathogenic	ID/MCA non syndromic	***
027	GDD, ALS	23	M	NAF	***	***	***	***	Negative	***	***
028	GDD, MS	2	M	Gain	0.16	arr[GRCh37] 8p23.3(1,586,553_1,748,941)x3 mat	*CLN8*	Inherited mat	VUS	***	***
029	GDD	4	F	Gain	0.40	arr[GRCh37] 1q21.1(145,382,123_145,786,290)x3 pat	*RBM8A, PEX11B*	Inherited pat	LP	Chromosome 1q21.1 duplication syndrome	612475
030	GDD,MS,MCA	11m	F	NAF	***	***	***	***	Negative	***	***
031	GDD	1	F	NAF	***	***	***	***	Negative	***	***
032	GDD	1	F	NAF	***	***	***	***	Negative	***	***
033	GDD	9	M	NAF	***	***	***	***	Negative	***	***
034	GDD	4	M	Loss	1.59	arr[GRCh37] 1p32.3(53,894,316_55,487,208)x1 dn	*DHCR24*	*de novo*	Pathogenic	Desmosterolosis	602398
035	GDD	16	F	NAF	***	***	***	***	Negative	***	***
036	GDD	15	M	Loss	2.88	arr[GRCh37] 22q11.21(18,916,842_1,800,797)x1 dn	*DGCR5, DGCR9, DGCR10, DGCR2, DGCR11, DGCR14, GSC2, CLTCL1, HIRA, MRPL40, CLDN5, GP1BB, TBX1, GNB1L, ARVCF, DGCR8, DGCR6L, ZNF74, MED15, SNAP29, LZTR1, SLC7A4*	*de novo*	Pathogenic	DiGeorge Syndrome	188400
037	MS,SUA	1m	F	NAF	***	***	***	***	Negative	***	***
038	GDD,MS	9	M	Gain	1.92	arr[GRCh37] 1q21.1q21.2(145,895,746_147,819,294)x3 pat	*HYDIN2*	Inherited pat	LP	ID/MCA non syndromic	***
				Gain	0.19	arr[GRCh37] 5p13.2(37,188,412_37,387,467)x3 pat	*C5orf42*	Inherited pat	LP		
039	GDD,MS	3	F	NAF	***	***	***	***	Negative	***	***
040	GDD	3	M	NAF	***	***	***	***	Negative	***	***
041	GDD,MS,MCA	2	F	Gain	0.16	arr[GRCh37] Xq26.3(134,687,985_134,848,809)x3 mat	***	Inherited mat	VUS	***	***
042	GDD, Microcephaly	6	F	Loss	8.2	arr[GRCh37] 6q26q27(162,708,065_170,919,482)x1 dn	*BRP44L, RNASET2, TBP*	*de novo*	Pathogenic	ID/MCA non syndromic	***

Supplementary table - Clinical and molecular features of patients investigated with CMA. (cont.)

Case	Clinical features*	Age (yo)	Sex	CNV	Size (Mb)	Microarray nomenclature	Selected OMIN Morbid Genes**	Origin	Interpretation	Syndrome	#OMIM
043	GDD	18	F	Loss	0.56	arr[GRCh37] 17q21.31(43,648,662_44,212,416)x1 dn	*KANSL1*	de novo	Pathogenic	Koolen-De Vries syndrome	610443
044	GDD,MS	16	M	Gain	0.17	arr[GRCh37] 9p13.3(34,211,171_34,385,700)x3 mat	***	Inherited mat	LP	ID/MCA non syndromic	***
045	GDD,MS,MCA,SS	15	M	NAF	***	***	***	***	Negative	***	***
046	GDD	1	F	NAF	***	***	***	***	Negative	***	***
047	GDD	18	F	Loss	0.28	arr[GRCh37] 21q11.2(15,006,457_15,285,841)x1 mat	***	Inherited mat	VUS	***	***
048	GDD	4	M	Gain	0.45	arr[GRCh37] 5q35.2q35.3(176,382,009_176,832,993)x3 dn	*NSD1*	*de novo*	Pathogenic	Sotos syndrome	117550
049	GDD,MS	13	M	Loss	0.12	arr[GRCh37] 6p25.3(254,253_375,828)x1 dn	***	*de novo*	LP	ID/MCA non syndromic	***
050	GDD	7	M	Loss	0.25	arr[GRCh37] 21q11.2(15,006,457_15,255,326)x1 dn	***	*de novo*	VUS	***	***
051	GDD,MS,MCA	2	F	NAF	***	***	***	***	Negative	***	***
052	GDD,MCA	10m	M	NAF	***	***	***	***	Negative	***	***
053	GDD	8	M	NAF	***	***	***	***	Negative	***	***
054	GDD,MS	10	M	Loss	0.13	arr[GRCh37] 3p24.2(26,039,618_26,170,698)x1 mat	***	Inherited mat	VUS	***	***
055	GDD	7	M	Gain	0.12	arr[GRCh37] 15q21.2(50,740,346_50,861,547)x3 mat	*USP8*	Inherited mat	VUS	***	***
056	GDD,DBEA,MCA	12	M	Loss	1.40	arr[GRCh37] 16p11.2(32,511,178_33,912,324)x1 dn	***	*de novo*	VUS	***	***
057	GDD	7	F	NAF	***	***	***	***	Negative	***	***
058	GDD	18	M	Gain	0.13	arr[GRCh37] 17q12(34,946,326_35,074,571)x3	***	NA	VUS	***	***
059	GDD	6	M	Loss	0.27	arr[GRCh37] 22q11.23q12.1(25,656,237_25,930,479)x1 dn	***	*de novo*	LP	Chromosome 22q11.2 deletion syndrome, distal	611867
060	GDD	11m	F	NAF	***	***	***	***	Negative	***	***
061	GDD,MCA	12	F	Gain	0.84	arr[GRCh37] Xp22.33(784,157_1,622,815)x3 mat	***	Inherited mat	VUS	***	***
062	GDD	12	F	NAF	***	***	***	***	Negative	***	***
063	GDD,MS,DBEA	22	F	Loss	0.51	arr[GRCh37] 17q21.31(43,703,801_44,212,416)x1 dn	*KANSL1*	*de novo*	Pathogenic	Koolen-De Vries syndrome	610443
064	GDD	4	F	Gain	0.69	arr[GRCh37] 22q11.22q11.23(22,962,196_23,652,512)x3 mat	***	Inherited mat	LP	ID/MCA non syndromic	***
065	GDD	9m	M	NAF	***	***	***	***	Negative	***	***
066	GDD,MS,BD	26	F	NAF	***	***	***	***	Negative	***	***
067	GDD,MS	2	M	NAF	***	***	***	***	Negative	***	***
068	GDD	10	M	NAF	***	***	***	***	Negative	***	***

Supplementary table - Clinical and molecular features of patients investigated with CMA. (cont.)

Case	Clinical features*	Age (yo)	Sex	CNV	Size (Mb)	Microarray nomenclature	Selected OMIN Morbid Genes**	Origin	Interpretation	Syndrome	#OMIM
069	GDD,MS	5	M	NAF	***	***	***	***	Negative	***	***
070	GDD	8	F	NAF	***	***	***	***	Negative	***	***
071	GDD,MS	2	F	Gain	0.90	arr[GRCh37] 3p26.3(585,118_1,480,555)x4 mat	*CNTN6*	Inherited mat	VUS	***	***
				Gain	0.11	arr[GRCh37] 12q24.22(117,648,219_117,753,672)x3 dn	*NOS1*	*de novo*	VUS		
072	GDD	14	F	NAF	***	***	***	***	Negative	***	***
073	GDD	6	M	NAF	***	***	***	***	Negative	***	***
074	GDD	23	M	Gain	0.23	arr[GRCh37] 10q26.3(135,024,951_135,252,347)x3	*ECHS1*	NA	VUS	***	***
				Loss	0.11	arr[GRCh37] 14q32.2(99,063,155_99,174,192)x1	***	NA	VUS		
075	GDD	17	F	NAF	***	***	***	***	Negative	***	***
076	GDD	10	M	NAF	***	***	***	***	Negative	***	***
077	GDD	12	F	NAF	***	***	***	***	Negative	***	***
078	GDD,MCA	8m	M	Gain	0.11	arr[GRCh37] 2p25.3(1,741,827_1,850,859)x3	*PXDN, MYT1L*	NA	VUS	***	***
079	GDD,MCA	68d	M	Gain	70.77	arr[GRCh37] 9p24.3q21.11(203,861_70,974,662)x4	9p24.3q21.11 tetrasomy	NA	Pathogenic	GDD/MCA non syndromic	***
080	GDD,MS,MCA,SS	10	M	Gain	12.9	arr[GRCh37] 11q12.1q13.3(56,928,391_69,867,039)x3	*TMEM216, SCYL1, NRXN2, RNASEH2C, PACS1, BBS1, SPTBN2*	NA	Pathogenic	Joubert syndrome 2	608091
081	GDD,DBEA	2	M	Gain	0.22	arr[GRCh37] Xq26.3(134,748,665_134,966,528)x2	***	NA	VUS	***	***
082	GDD,MS	26a	F	Loss	2.5	arr[GRCh37] 22q11.21(18,916,842_21,465,659)x1	*DGCR5, DGCR9, DGCR10, DGCR2, DGCR11, DGCR14, GSC2, CLTCL1, HIRA, MRPL40, CLDN5, GP1BB, TBX1, GNB1L, ARVCF, DGCR8, DGCR6L, ZNF74, MED15, SNAP29, LZTR1 e SLC7A4*	NA	Pathogenic	DiGeorge Syndrome	188400
083	GDD, MS	7a	F	Gain	1.75	arr[GRCh37] 22q11.1q11.21(16,888,899_18,644,773)x4	*CECR7, CECR6, CECR5, CECR1, CECR3, CECR2, SLC25A18*	NA	Pathogenic	Chromosome 22q11.2 duplication syndrome	608363
084	GDD, SS	2a	F	Loss	1.99	arr[GRCh37] 15q26.3(100,432,397_102,429,112)x1	*ADAMTS17, LINS1*	NA	Pathogenic	GDDD/MCA non syndromic	***

* SS=Short Stature; GDD=Global Developmental Delay; MS=Multiple Stigmas, MCA=Multiple Congenital Anomalies, ALS= Autism Like Symptoms; BD=Behavior Disorders; DBEA=Disturbance of brain electrical activity; SUA=Single Umbilical Artery; LP=likely pathogenic; VUS= Variant of uncertain significance; NA= No available; ** Genes related to ID/Autism; *** Not applicable.
